# Mitochondrial DNA Mutations as a Factor in the Heritability of Atherosclerosis and Other Diseases

**DOI:** 10.2174/0109298673291199241129044139

**Published:** 2025-01-14

**Authors:** Alexander N. Orekhov, Nikolay A. Orekhov, Vasily N. Sukhorukov, Victoria A. Khotina, Tatiana I. Kovianova, Igor A. Sobenin

**Affiliations:** 1 Laboratory of Angiopathology, Institute of General Pathology and Pathophysiology, 8, Baltiiskaya Street, Moscow, 125315, Russia;; 2 Petrovsky Russian National Center of Surgery, Insitute of Human Morphology, Abrikosovsky Lane, Moscow, 119991, Russia;; 3 National Medical Research Center of Cardiology, 15a, Academician Chazov Street, Moscow, 121552, Russia

**Keywords:** Atherosclerosis, low-density lipoprotein, innate immune system, inflammatory reaction, intolerant immune response, cybrid, genome editing, chronification of inflammation, mitochondrial dysfunctions, mitochondrial DNA mutations

## Abstract

This review discusses the possibility of inheritance of some diseases through mutations in mitochondrial DNA. These are examples of many mitochondrial diseases that can be caused by mutations in mitochondrial DNA. Symptoms and severity can vary widely depending on the specific mutation and affected tissues. An association between certain mutations in the mitochondrial genome and cancer was reported. In other studies of 2-4 generations in each family, we found that mitochondrial mutations associated with atherosclerosis are inherited. This may at least partially explain the inheritance of predisposition to atherosclerotic disease by maternal line. Furthermore, to prove the important role of mitochondrial mutations in the development of atherosclerotic manifestations at the cellular level, we developed a technique for editing the mitochondrial genome. A recent article described how one of the pro-atherogenic mutations, namely m.15059G>A, was eliminated from such monocyte-derived cells using the technique we developed. Elimination of this mutation resulted in the restoration to normal levels of initially defective mitophagy and impaired inflammatory response. These data strongly suggest that mitochondrial mutations are closely associated with the development of atherosclerotic lesions. Considering that they are inherited, it can be assumed that, at least partly, the genetic predisposition to atherosclerotic diseases is transmitted from mother to offspring. Thus, despite the small size of mitochondrial DNA, its mutations may play a role in the pathogenesis of diseases. Further study of their role will make it possible to consider mitochondrial mutations as promising diagnostic markers and disorders caused by mutations as pharmacological targets.

## 1 INTRODUCTION

The journal has published couple of dozen articles related to mitochondrial dysfunctions [1-6]. The growing interest in this topic is explained by the serious and sometimes even key role of mitochondrial dysfunctions in various pathologies [7-16]. Mitochondrial dysfunctions are the result primarily of two causes, namely: (i) mitochondrial DNA (mtDNA) mutations and (ii) mitochondrial respiratory chain disorders. Mutations in the mitochondrial DNA can lead to dysfunction in the mitochondria, affecting their ability to produce energy efficiently [17-20]. These are a group of disorders that affect the electron transport chain in the mitochondria, leading to impaired energy production [21, 22]. In addition to spontaneous somatic mtDNA mutations, mitochondrial dysfunction can be caused by a variety of other factors, including:

- The damage to the nuclear and mitochondrial genome. Inherited mutations in the mitochondrial DNA or nuclear DNA can lead to mitochondrial dysfunction [23-30]. These mutations can affect the structure or function of the mitochondria.

### 1.1 Environmental Factors

Exposure to toxins, pollutants, and certain medications can damage the mitochondria and disrupt their function [31-40]. Oxidative stress, which occurs when there is an imbalance between free radicals and antioxidants in the body, can also contribute to mitochondrial dysfunction [41-48].

- Age-related changes. Mitochondrial function naturally declines with age, leading to decreased energy production and increased oxidative damage. This age-related decline in mitochondrial function can contribute to various health problems associated with aging [49-56].

- Metabolic disorders. Such conditions as diabetes, obesity, and metabolic syndrome can affect mitochondrial function and lead to dysfunction. Insulin resistance, inflammation, and abnormal lipid metabolism associated with these conditions can impact mitochondrial health [57-60].

### 1.2 Nutritional Deficiencies

Inadequate intake of essential nutrients, such as vitamins, minerals, and antioxidants, can impair mitochondrial function. Mitochondria require a variety of nutrients to function properly, and deficiencies can disrupt their energy production and overall health [61-65].

- Mitochondrial biogenesis. Disruption in the process of mitochondrial biogenesis, which involves the creation of new mitochondria, can lead to mitochondrial dysfunction. Factors that regulate mitochondrial biogenesis, such as exercise, calorie restriction, and certain signaling pathways, can influence mitochondrial health [66-68].

### 1.3 Mitochondrial Dynamics

Imbalances in mitochondrial fusion and fission processes, which control the shape and distribution of mitochondria within cells, can also contribute to mitochondrial dysfunction. Disruption in these processes can affect mitochondrial function and cellular energy metabolism [69-71].

Mitochondrial mutations, if inherited, may explain the heritability of at least some diseases. It has been reported that mitochondrial mutations can lead to a group of disorders known as mitochondrial diseases (cytopathies). These diseases can affect various organs and systems in the body. Below is a list of some common mitochondrial diseases caused by mutations in mitochondrial DNA.

### 1.4 Mitochondrial Myopathy

This condition affects the muscles and can cause weakness, exercise intolerance, and muscle pain [72-74].

Leber's hereditary optic neuropathy (LHON): LHON is a type of mitochondrial disease that primarily affects the optic nerve, leading to vision loss [75, 76].

- Kearns-Sayre syndrome: This is a rare mitochondrial disorder that affects multiple systems in the body, including the eyes, heart, and muscles [77, 78].

- MELAS syndrome (Mitochondrial Encephalomyopathy, Lactic Acidosis, and Stroke-like episodes): This condition can cause a variety of symptoms, including muscle weakness, seizures, stroke-like episodes, and lactic acidosis [79, 80].

- Leigh syndrome: Leigh syndrome is a severe neurological disorder that typically presents in infancy or early childhood and can lead to developmental delays, movement disorders, and respiratory issues [81, 82].

These are just a few examples of many mitochondrial diseases that can be caused by mutations in mitochondrial DNA. Symptoms and severity can vary widely depending on the specific mutation and affected tissues [83-85].

In a recently published paper by Funke *et al.* [86], the authors found an association between certain mutations in the mitochondrial genome and cancer. Previously, we have identified the association of certain mitochondrial mutations (genome variants) with atherosclerosis [87]. These mutations are inherited and affect cell function (Table **1**) [88].

Maternal relatives were examined in 31 families (1 family - 4 generations, 21 families - 3 generations, 7 families - 2 generations). Monocytes were isolated from the mononuclear fraction by affinity separation using magnetic CD14-affinity microparticles on LS Separation Columns (Miltenyl Biotec, Germany). The mutational load of the mitochondrial genome was estimated by mutations m.3256C>T, m.13513G>A, m.12315G>A, and m.15059G>A. The choice of mutations for the study was due to their high occurrence and the level of heteroplasmy, which is sufficient for evaluation in the mathematical model of mutation inheritance. The heteroplasmy of the mitochondrial genome based on these mutations was assessed by pyrosequencing of short-chain mtDNA fragments. According to the analysis, the probability of the hereditary nature of the m.3256C>T mutation was 83%, the m.13513G>A mutation was 98%, the m.12315G>A mutation was 98%, and the m.15059G>A mutation was 92%. We also assessed the probability that the studied mutations occur sporadically in any generation and are further transmitted through the maternal line and accumulate from generation to generation. This probability for the m.3256C>T mutation was 38%, the m.13513G>A mutation was 20%, the m.12315G>A mutation was 25%, and the m.15059G>A mutation was 25%.

As shown in Table **1**, the probability of the hereditary nature of the above 4 mitochondrial DNA mutations is several-fold higher than the possibility of their sporadic occurrence. Furthermore, this may explain the preservation of such mutations in a population. Moreover, considering their association with atherosclerosis [89], this may partially explain the inheritance of predisposition to atherosclerotic disease through the maternal line.

We are studying the mechanisms of the relationship between atherosclerosis and mitochondrial mutations [88].

**Fig. (1):**
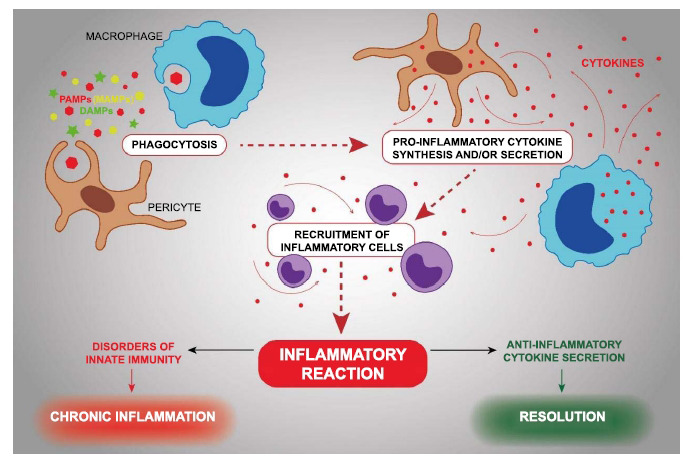
Role of inflammation in initial stages of atherosclerosis.

The very initial stages of atherosclerosis are accompanied by local accumulation of intra- and extracellular lipids in the intima (the inner layer closest to the lumen) of the arteries (Fig. **1**). The source of these lipids is low-density lipoprotein (LDL) circulating in the blood [90]. Moreover, lipoprotein particles that cause lipid accumulation differ from native LDL in many chemical and physical changes [90]. Such modified LDL particles can develop as damage-associated molecular patterns (DAMPs), which, along with pathogen-associated molecular patterns (PAMPs), trigger phagocytosis of intimal subendothelial cells, which is the initial stage of the innate immune response. In the intima, the main phagocytic cells are macrophages and macrovascular pericytes [91]. In addition, smooth muscle cells can capture LDL and accumulate intracellular lipids, but to a much lesser extent than “professional” phagocytes. Phagocytosis is accompanied by the secretion of cytokines, which promote further accumulation of lipids and cause the recruitment of immune cells to the site of inflammation [92].

Damage-associated molecular patterns (DAMPs), including modified LDL-causing accumulation of intracellular lipids, as well as pathogen-associated molecular patterns (PAMPs), trigger phagocytosis of intimal subendothelial cells. Phagocytosis stimulates the secretion of proinflammatory cytokines, which attract immune (inflammatory) cells to the emerging site of inflammation. Inflammation more or less quickly ends with resolution. If, at the level of innate immunity, the inflammation machinery is damaged, the acute form of inflammation becomes chronic, which accompanies the development of atherosclerotic lesions at all subsequent stages.

**Fig. (2):**
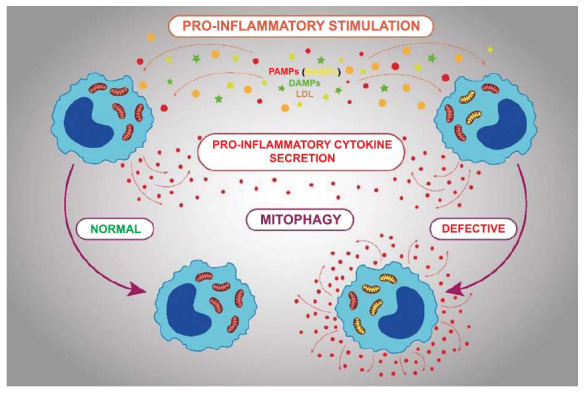
Mitochondrial mutations, defective mitophagy and inflammation.

The inflammatory reaction with a normally functioning innate immune system resolves quickly [XX]. However, when the immune response is impaired, rapid resolution does not occur, and the inflammatory reaction becomes chronic (Fig. **2**). We have shown that one of the causes of chronification of inflammation may be mitochondrial mutations [88]. In particular, it has been shown that defective mitophagy occurs in cells carrying pro-atherogenic mitochondrial mutations, that is, mitophagy stimulators have no effect in these cells. It was in these cells with defective mitophagy that an impaired immune response was identified [93]. Impaired immune response manifested itself in intolerant secretion of proinflammatory cytokines in response to inflammatory stimulation. In a normal immune response, repeated inflammatory stimulation of cells causes lower secretion of cytokines compared to the first stimulation, which leads to a gradual attenuation of the immune response and, ultimately, to the resolution of inflammation [94]. With an intolerant immune response, repeated inflammatory stimulation causes the same cytokine secretion as the first stimulation or even higher [95]. As a result, the immune response remains incomplete but develops continuously, and the inflammation does not resolve. Thus, the inflammation becomes chronic.

Mitochondria carrying pro-atherogenic mutations are characterized by defective mitophagy. Cells with such mitochondria exhibit an impaired proinflammatory response, expressed in the persistent secretion of proinflammatory cytokines. At the same time, cells with normal mitophagy gradually stop releasing cytokines, and the cell ceases to be a source of pro-inflammatory signals.

**Fig. (3):**
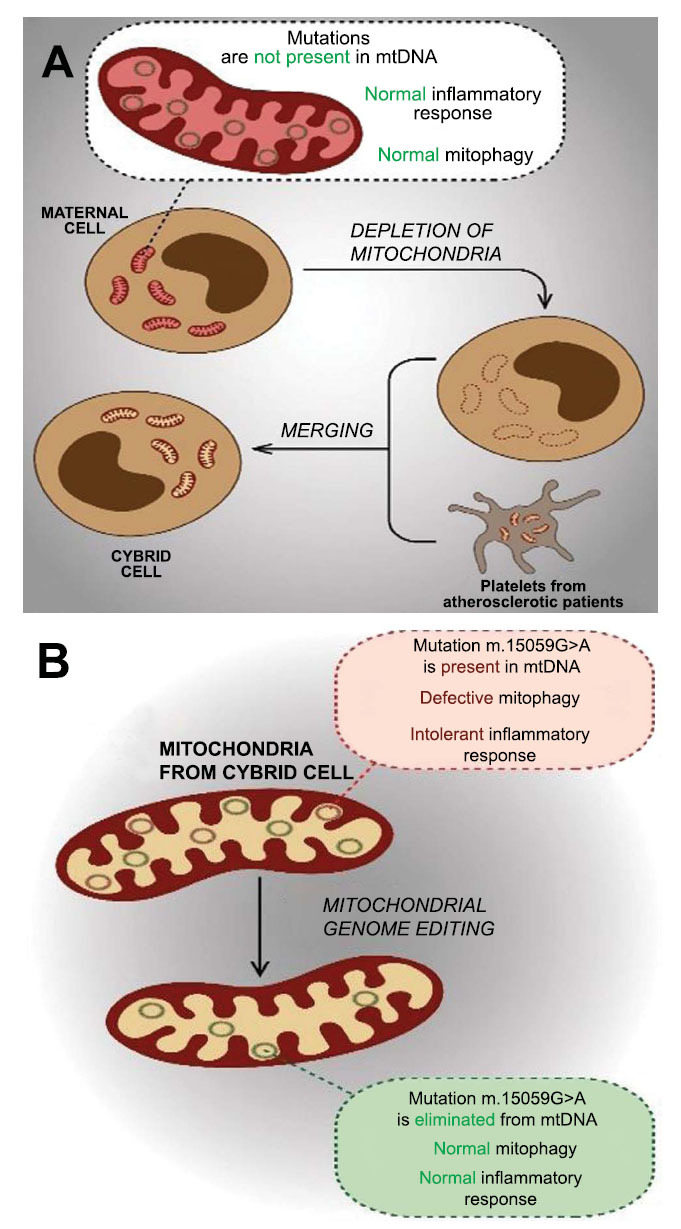
Evidence of the important role of mitochondrial mutations in the development of atherosclerotic manifestations at the cellular level. (**A**) Cytoplasmic hybrids (cybrids) were obtained by fusion of mitochondria-free linear cell THP-1 and platelets from atherosclerotic patients. The result was a linear cell carrying mitochondrial mutations associated with atherosclerosis, in particular m.15059G>A. The mitochondria of such a cell exhibit defective mitophagy and intolerant response to inflammatory stimulation. (**B**) Using a specially developed mitochondrial genome editing technique, mitochondrial DNA with the m.15059G>A mutation was removed from the cell. This led to the restoration of normal mitophagy and a normal (tolerant) inflammatory response.

Furthermore, to prove the important role of mitochondrial mutations in the development of atherosclerotic manifestations at the cellular level, we developed a technique for editing the mitochondrial genome [96]. The work used cytoplasmic hybrids (cybrids) obtained by merging a maternal linear cell deprived of its own mitochondria with platelets from atherosclerotic patients. Such cybrids have a maternal cell nucleus, but in the cytoplasm, there are mitochondria from the cells of atherosclerotic patients. Unlike the mother cell, the cybrid contains pro-atherogenic mitochondrial mutations; most mitochondria exhibit defective mitophagy, which is associated with an intolerant inflammatory reaction of the cybrid (Fig. **3**). One of the pro-atherogenic mutations, namely m.15059G>A, was eliminated from such a cybrid using the technique we developed. Elimination of this mutation resulted in the restoration of normal mitophagy and a normal (tolerant) inflammatory response (Fig. **3**) [96]. These data strongly suggest that mitochondrial mutations are closely associated with the development of atherosclerotic lesions. Considering that they are inherited, it can be assumed that, at least partly, the genetic predisposition to atherosclerotic diseases is transmitted from mother to offspring.

Fig. (**4**) schematically outlines the involvement of mitochondrial DNA mutations in atherogenesis. LDL circulating in the blood of patients undergoes multiple atherogenic modifications [90]. Modified LDL particles form self-associates that are taken up by subendothelial cells through phagocytosis [97, 98]. Phagocytosis triggers an innate immune response that manifests itself in the secretion of proinflammatory cytokines, which are a signal for the recruitment of immune cells to the site of inflammation [99]. The inflammatory reaction does not last long and ends with resolution. A slight compaction of tissue occurs at the site of inflammation. Similar foci of inflammation appear in the intima here and there over time, resulting in diffuse thickening of the intima characteristic of an adult. Diffuse intimal thickening is not an atherosclerotic lesion [100, 101].

The top panel demonstrates a favorable development of events. LDL circulating in the blood undergoes modification, which leads to the appearance of atherogenic properties, manifested in the ability to cause the accumulation of lipids in arterial cells. Modified LDL penetrates into the intima and enters the arterial cell by phagocytosis, which triggers an innate immune response accompanied by the secretion of pro-inflammatory cytokines that attract immune (inflammatory) cells that trigger an inflammatory reaction in which mitochondria are actively involved. If there are no pro-atherogenic mutations in the mitochondrial DNA, the mitochondria will function normally, and the inflammation will resolve fairly quickly. At the site of the former focus of inflammation, a compaction with a slight thickening (micro-scar) appears. Over time, such micro-scars form diffuse intimal thickening, which is not an atherosclerotic lesion but a normal age-related change.

The bottom panel demonstrates an unfavorable development of events (formation of atherosclerotic lesions). Pro-atherogenic mitochondrial mutations in resident intimal cells and/or recruited immune cells can cause mitochondrial dysfunction manifested in particular by defective mitophagy, which prevents the elimination of dysfunctional mitochondria. Such mitochondria will be a constant intracellular trigger for the synthesis and secretion of proinflammatory cytokines. The inflammation does not resolve, and it becomes chronic. Local chronic inflammation in the arterial wall is the cause of the appearance of initial atherosclerotic lesions and accompanies the development of severe lesions that pose a threat to health and life.

## CONCLUSION

A different picture is observed if the subendothelial cell carries pro-atherogenic mutations in mitochondrial DNA, which are associated with defective mitophagy, which in turn disrupts the innate immune response manifested in intolerant secretion of cytokines. Such a cell will continuously secrete much more pro-inflammatory cytokines than a cell that does not have pro-atherogenic mutations [102]. More and more immune cells are recruited to the site of inflammation, some of which may carry pro-atherogenic mutations of mitochondrial DNA, which will aggravate the chronification of inflammation. Local chronic inflammation of the arterial wall can last for years and manifests itself in the form of a pronounced atherosclerotic plaque, which poses a danger to the health and life of the patient.

Thus, despite the small size of mitochondrial DNA, its mutations may play a role in the pathogenesis of diseases. Further study of their role will make it possible to consider mitochondrial mutations as promising diagnostic markers and disorders caused by mutations as pharmacological targets.

## Figures and Tables

**Fig. (4):**
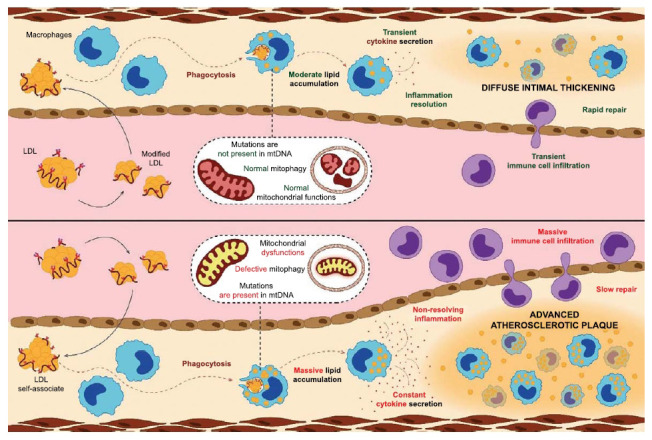
The involvement of mitochondrial DNA mutations in atherogenesis.

**Table 1:** Inheritance of mitochondrial DNA mutations.

**Mutation**	**Probability of the Hereditary Nature of the Mutation**	**Probability of the Sporadic Occurrence of the Mutation**
m.3256C>T	83%	38%
m.13513G>A	98%	20%
m.12315G>А	98%	25%
m.15059G>A	92%	25%

## LIST OF ABBREVIATIONS

DAMPs: Damage-associated Molecular Patterns
DNA: Deoxyribonucleic Acid
LDL: Low-density Lipoprotein
LHON: Leber's Hereditary Optic Neuropathy
MELAS: Mitochondrial Encephalomyopathy, Lactic Acidosis, and Stroke-like Episodes
PAMPs: Pathogen-associated Molecular Patterns
